# Low-Dose Apalutamide in Nonmetastatic Castration-Resistant Prostate Cancer: A Case Report

**DOI:** 10.7759/cureus.54197

**Published:** 2024-02-14

**Authors:** Minh Dung Nguyen, Gilles Natchagande, Olena Gorobets, Vincent Vinh-Hung

**Affiliations:** 1 Orthopedics and Sport Medicine, Hospital of Orthopedics and Rehabilitation, Ho Chi Minh City, VNM; 2 Urology, Centre Hospitalier Universitaire Départementale Ouémé Plateau (CHUD-OP) and Centre National Hospitalier Universitaire Hubert Koutoukou MAGA (CNHU-HKM), Cotonou, BEN; 3 Radiation Oncology, Centre Hospitalier Universitaire (CHU) de Martinique, Fort-de-France, FRA; 4 Radiation Oncology, Universitair Ziekenhuis (UZ) Brussel, Brussels, BEL; 5 Radiation Oncology, Institut Bergonié, Bordeaux, FRA

**Keywords:** nonmetastatic castration-resistant prostate cancer, castration resistant prostate cancer, prostate cancer, apalutamide, androgen receptor inhibitor

## Abstract

The effect of low-dose apalutamide in nonmetastatic castration-resistant prostate cancer is unknown. We report the observation of therapy being administered at 25% of the recommended dose in an 80-year-old patient. Despite treatment discontinuation during COVID lockdowns, he survived three years without evidence of metastasis. This case gently invites us to reflect on the possibility of low-dose apalutamide in the elderly.

## Introduction

In 2020, prostate cancer was the third most common newly diagnosed cancer among males in Europe, with approximately 470,000 new cases reported [1]. Nonmetastatic castration-resistant prostate cancer (nmCRPC) is a transient disease state characterized by the observation of increasing prostate-specific antigen (PSA) levels, despite a patient undergoing androgen deprivation therapy, and the absence of detectable metastases based on conventional imaging, such as computer scans, technetium-99m scintigraphy, and gallium-68 prostate-specific membrane antigen (Ga-68 PSMA) ligand scintigraphy [2]. A PSA doubling time of less than 10 months in nmCRPC serves as a good prognostic indicator for disease progression, significantly predicting patient survival outcomes [3]. In 2019, there were following two options for androgen deprivation therapy for nmCRPC: enzalutamide and apalutamide [4]. In France, apalutamide was approved for reimbursement in nmCRPC in 2019, enzalutamide soon after. Apalutamide demonstrates efficacy in the treatment of nmCRPC [5]. Although current evidence supports using a 240 mg dose of apalutamide for nmCRPC, we reasoned by analogy that a reduced dosage of 60 mg/d, or 25% of the recommended dose, could be considered in the elderly patient, as had been done with enzalutamide [6]. The structural similarity between apalutamide and enzalutamide, along with comparable adverse effect profiles, suggested that low-dose apalutamide could be as efficient as conventional-dose apalutamide, the same way that low-dose enzalutamide was found as efficient as standard-dose enzalutamide in the elderly [7]. By sharing a retrospective case, for which informed consent has been obtained, we aimed to provide valuable insights gained from treating an elderly man who had nmCRPC.

## Case presentation

The patient, who had no prior history of disease, attended an oncology consultation for a nonmetastatic locally recurrent prostate cancer. He was diagnosed at the age of 67.3 years with prostate adenocarcinoma and showed the following characteristics: Gleason score 7 (4+3), initial PSA 30.6 ng/mL, and clinical stage T3aN0M0 (Table 1). He was reported as having severe obstructive urinary symptoms. The patient received radiotherapy of 46 Gy to the pelvic lymph nodes and of 74 Gy to the prostate and the base of the seminal vesicles. Testosterone suppression by luteinizing hormone-releasing hormone analog (LHRHA) started five months before radiotherapy and continued for three years.

**Table 1:** Patient characteristics and comparison with those reported in the SPARTAN trial. PSA: prostate-specific antigen; SPARTAN: Selective Prostate AR Targeting with ARN-509; LHRH: luteinizing hormone-releasing hormone; ECOG: Eastern Cooperative Oncology Group

Characteristic		This case	Trial SPARTAN [5]
Age at initial diagnosis (years)	67.30		-
Weight at diagnosis (kg)	63		-
Weight at start apalutamide (kg)	76		-
Gleason score	7		-
Initial PSA (ng/mL)	30.6		-
Clinical stage	T3aN0M0		-
First therapy	Radiation + hormone		-
Castration status at start of apalutamide	Yes		-
Metastases	No		-
Dose apalutamide (mg)	60		240
Age at start apalutamide (years)	80.20		74
Age at last follow-up (years)	83.62		Estimated: 80.16
Length of survival from start apalutamide to death	1,252 days = 44.7 months		Median overall survival = 73.9 months = 6.16 years
Treatment duration	1,252 days minus 6 months interruption = 38.7 months		32.9 months
ECOG at start apalutamide	2		0
Pain score at start apalutamide (range 0-10)	0		-
Last known pain score (range 0-10)	3		-
Last known weight (kg)	75		-
ECOG at 2 years	0		-
Nadir PSA (ng/mL)	3.98		-
Therapy before apalutamide	bicalutamide + LHRH-agonist		-
Testosterone at start apalutamide (ng/mL)	0.18		-

The patient achieved an asymptomatic state with a favorable PSA level and sustained complete remission until he reached the age of 76.5 years. At that time, local prostate recurrence with extracapsular extension was diagnosed on magnetic resonance imaging; there was no distant metastatic localization on computed tomography or bone scan. Renewed testosterone suppression with intramuscular triptorelin 22.5 mg injection every six months started at the age of 77.3 years.

Subsequent assessments of serum testosterone consistently indicated that optimal castration levels had been achieved, with a recorded decline of testosterone to 0.18 ng/mL at 79.9 years of age. The PSA level fluctuated and then increased to 15.31 ng/mL at 80.2 years. The estimated PSA doubling time was two months. Computed tomography and bone scan at that time again showed no metastatic disease.

The patient was considered eligible for apalutamide therapy. At clinical evaluation, he had an Eastern Cooperative Oncology Group (ECOG) performance status of 2. He had walking instability, was visually impaired, and required help for daily activities; otherwise, he had no known comorbidities, no complaint, and no urinary symptoms other than two or three nightly mictions. Apalutamide was prescribed at a daily dose of 60 mg, instead of the recommended 240 mg, with continued LHRHA.

The first PSA control at two months showed a response, with the PSA level dropping to 6.54 ng/mL. COVID-19 lockdown intervened, and the patient was not seen again until he was 81.2 years old. The PSA had increased to 26.67 ng/mL, and we learned that his supply of apalutamide had been discontinued for six months. It was consequently renewed, and his PSA level dropped to 3.98 ng/mL after six months, with a testosterone level of 0.31 ng/mL. Phone calls to his nurse indicated that he had help taking the medication. At the age of 82.53 years, the patient’s PSA level was 17.94 ng/mL and testosterone was 0.26 ng/mL. He had no pain or symptoms of adverse effects. After multidisciplinary discussion among his care providers, new imaging studies and an increased dosage of apalutamide to 120 mg were planned, although bone scan and computed tomography showed no evidence of metastasis.

We lost contact with the patient and learned later that he had experienced a cerebral vascular accident. Owing to the patient’s debilitated condition, use of a permanent urine catheter, and 25 kg weight loss, all anti-cancer therapy was discontinued. The patient died at the age of 83.7 years (Table 2). His family and general practitioner informed us that there was no sign of cancer progression.

**Table 2:** PSA values during treatment periods. LHRHA: luteinizing hormone-releasing hormone analog; PSA: prostate-specific antigen

Consultation timepoint (age, years)	PSA (ng/mL)	On therapy	Metastasis on imaging
67.3	30.6	Started radiotherapy and LHRHA for 3 years	No
76.5	3.41	-	-
77.3	-	Restarted on triptorelin	-
78.2	0.15	-	-
79.4	0.26	-	-
79.9	0.18	-	-
80.2	15.31	Started on apalutamide 60 mg	No
80.4	6.54	-	-
81.2	26.67	-	-
81.7	3.98	-	-
82.65	17.94	Planned for apalutamide 120 mg	No
83.7	-	Died	-

## Discussion

This case highlights the challenges in the management of frail geriatric patients. A real-world retrospective study of apalutamide in nmCRPC reported on patients with an estimated median age of 80 years, comparable to our case, but with a shorter follow-up (median one year) and a mean duration of apalutamide treatment of 259 days [8]. Although our patient had no co-morbidities, he was not autonomous and was highly dependent on nursing help. Several consultations were canceled, and COVID-19 led to a lack of adherence to treatment. No studies have shown an increased risk of cerebral vascular accident in a patient using apalutamide.

**Figure 1:**
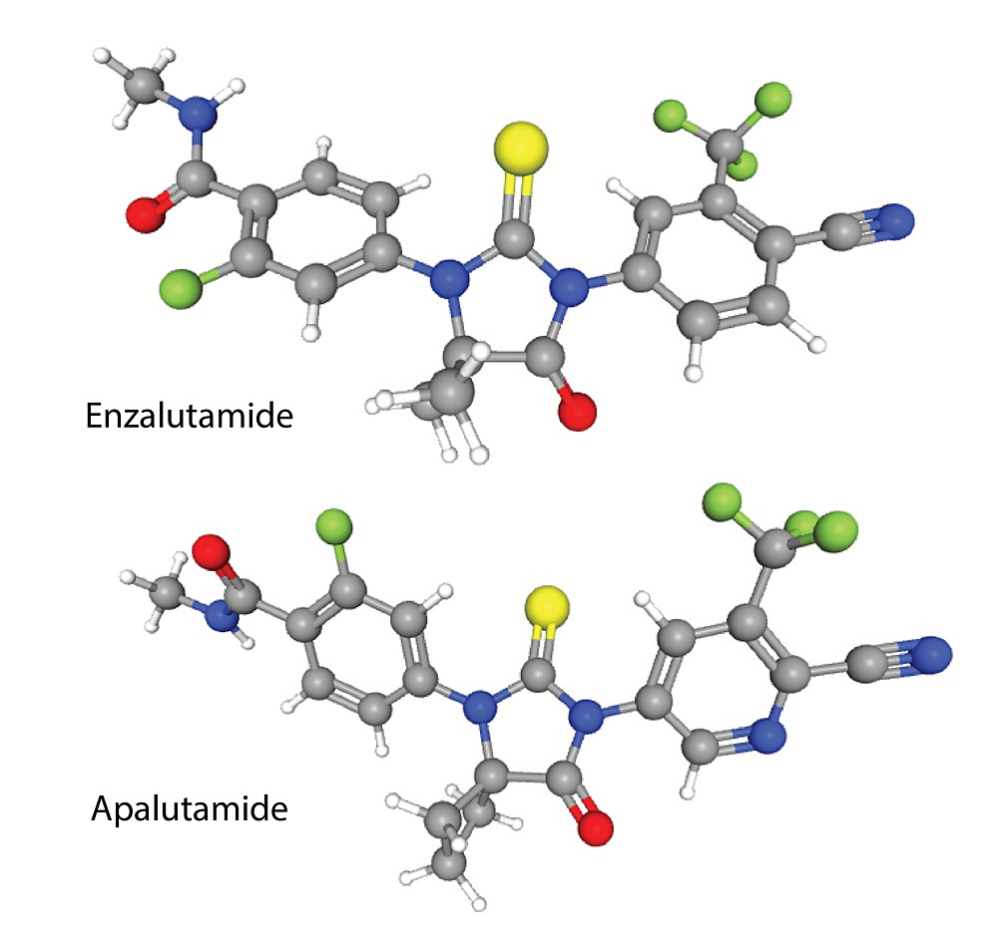
Structure of enzalutamide and apalutamide. Three-dimensional view drawn from PubChem (https://pubchem.ncbi.nlm.nih.gov), a public chemical database at the National Center for Biotechnology Information (NCBI) of the National Library of Medicine (NLM), an institute within the U.S. National Institutes of Health (NIH).

Apalutamide is associated with a good response to treatment, leading to a longer time of metastasis-free survival for patients with nmCRPC compared with a placebo group in a previous randomized controlled trial study [9]. In that trial, the overall quality of life was preserved in patients receiving the medication [10]. In the current case, there was a six-month interruption in treatment with apalutamide, but it did not appear to change the efficacy of low-dose apalutamide on PSA response. There was no pain, rash, or other common adverse effects in this case [9]. Response to low-dose apalutamide in the patient is in line with our earlier observation with enzalutamide, concordant with the similarity of the molecules (Figure 1) [7].

Compared with results from the Selective Prostate AR Targeting with ARN-509 (SPARTAN) study, despite poorer ECOG, higher PSA, and a lower starting dose of apalutamide, treatment duration was longer even after accounting for the COVID-19 discontinuation (Table 1). The SPARTAN apalutamide patients’ population had longer overall survival, but started at a younger age (74 years) and died 3.4 years sooner (a survival period of 6.2 years, resulting in a total lifespan of 80.2 years) as compared with the patient’s lifespan of 83.6 years (Table 1) [5].

The last increase in our patient’s PSA level suggested that the apalutamide dose was insufficient. The multidisciplinary team proposed increasing it, but without assurance that he would have help taking the medication, it is unclear whether increasing the prescribed dose would have made sense given his visual impairment. Blood sampling to monitor apalutamide concentration could help in such situations.

Although there is no published prospective study of low-dose apalutamide, NCT04530552 is an ongoing prospective phase 2 clinical trial that is expected to reach completion soon, in August 2024. NCT04530552 tests the effect of very low doses of apalutamide given before surgery for localized prostate cancer. This is an important study that will give precious information on the efficiency of very low doses on PSA and on surgical pathology outcomes [11].

## Conclusions

Using low-dose apalutamide for an elderly individual with a poor ECOG status could potentially extend metastasis-free periods, minimize adverse events for the patient, and prolong PSA doubling times. In the current case, the efficacy of low-dose apalutamide appeared to persist even after a six-month interruption. Results of NCT04530552 will be important to determine if extended studies with large sample sizes are warranted.
